# Culturally Adapting a Transdiagnostic Problem-Solving Intervention for British Bangladeshi Youth: A Qualitative Co-design Study

**DOI:** 10.1192/j.eurpsy.2026.10550

**Published:** 2026-07-31

**Authors:** I. Shahnaz, P. C. Gronholm, E. Cini, D. Michelson

**Affiliations:** 1 Department of Child and Adolescent Psychiatry, King’s College London, London, United Kingdom; 2 Department of Psychology, University of Dhaka, Dhaka, Bangladesh; 3 Centre for Global Mental Health, Department of Population Health, London School of Hygiene & Tropical Medicine; 4 Department of Child and Adolescent Psychiatry, King’s College London; 5 East London NHS Foundation Trust; 6 Nutrition Science Group, Division of Medicine, University College London; 7 NIHR Maudsley Biomedical Research Centre, South London and Maudsley NHS Foundation Trust and King’s College London, London, United Kingdom

## Abstract

**Introduction:**

Young British Bangladeshis experience a higher prevalence of mental health problems than their White British peers, yet are underrepresented in research and have less access to services. Cultural dissonance between Bangladeshi values and British norms, especially during the key developmental stage of 16–24 years, can intensify identity conflict and emotional distress. Despite these risks, uptake of talking therapies amongst this group remains low, potentially due to mistrust of formal services and reliance on informal support. This underutilisation of services highlights the need for culturally sensitive interventions that reflect the lived experiences of this population. Problem-solving therapy (PST), a structured, easily teachable cognitive-behavioural approach rooted in Lazarus and Folkman’s (1984) stress-coping model, offers a promising solution. PST promotes proactive coping by building stepwise problem-solving skills and has proven effective in diverse, low-resource settings. Its concreteness and flexibility appeal to youth, making it well suited for adaptation in high-income, multicultural contexts.

**Objectives:**

This study aims to culturally adapt a problem-solving intervention, originally developed in India, for British Bangladeshi youth through a participatory co-design process to improve its relevance, accessibility, and acceptability.

**Methods:**

This study includes two co-design workshops with 32 British Bangladeshi youth (aged 16–24) to ideate and test the intervention, with prototyping taking place between the two workshops. The first workshop (ideate phase) was carried out (April 2025), involving seven focus group discussions with young people, generating key insights and ideas for the intervention. The ongoing (summer 2025) prototype phase involves iterative development of intervention materials in collaboration with a youth co-researcher. The user testing phase, scheduled for later in 2025, will assess the acceptability and usability of the prototype through further focus groups and observations.

**Results:**

Using the Ecological Validity Model (EVM), adaptations were identified across all eight dimensions: culturally appropriate language; preferred types of counsellor; culturally resonant metaphors; content grounded in lived experience; cultural framing of mental health and problem-solving steps; outcomes aligned with PST goals; delivery strategies suited to youth needs; and relevant community settings. These insights are shaping the intervention prototype.

**Conclusions:**

This study shows how a problem-solving intervention can be co-designed with young people using participatory methods. It will yield a culturally tailored intervention for British Bangladeshi youth, with potential to increase engagement with evidence-based mental health support.

**Disclosure of Interest:**

None Declared

